# Utility of hydroxyurea in mast cell activation syndrome

**DOI:** 10.1186/2162-3619-2-28

**Published:** 2013-10-09

**Authors:** Lawrence B Afrin

**Affiliations:** 1 Division of Hematology/Oncology, Medical University of South Carolina, Charleston, South Carolina, USA

**Keywords:** Mast cell activation disease, Mast cell activation syndrome, KIT mutations, Pain, Hydroxyurea

## Abstract

Mast cell activation syndrome (MCAS) is a relatively recently recognized cause of chronic multisystem polymorbidity of a generally inflammatory theme. Patients with MCAS often report migratory soft tissue and/or bone pain which frequently responds poorly to typical (narcotic and non-narcotic) analgesics as well as atypical analgesics such as antidepressants and anticonvulsants. Hydroxyurea (HU) is an oral ribonucleotide reductase inhibitor commonly used in the treatment of chronic myeloproliferative neoplasms and sickle cell anemia. HU has been used to treat systemic mastocytosis, sometimes effecting improvement in MC activation symptoms but not tumor burden, suggesting potential utility of the drug in MCAS, too. Reported here are five cases of successful use of relatively low-dose HU in MCAS to reduce symptoms including previously refractory soft tissue and/or bone pain. HU may be useful in treating mediator symptoms in MCAS, but further study is needed to define optimal dosing strategies and patient subpopulations most likely to benefit.

## Introduction

Mast cell activation syndrome (MCAS, a more prevalent but only recently recognized cousin of the rare, proliferative mast cell (MC) disease mastocytosis) typically causes chronic multisystem polymorbidity of a generally inflammatory theme [1]. The MC activation seen in either mastocytosis or relatively non-proliferative MCAS often results in migratory soft tissue and/or bone pain which frequently responds poorly to typical (narcotic and non-narcotic) analgesics as well as atypical analgesics such as antidepressants and anticonvulsants.

Hydroxyurea (HU) is an oral ribonucleotide reductase inhibitor with antimetabolic and antineoplastic properties [2]. First used clinically in the 1960s for chronic myeloproliferative neoplasms (MPNs) [3], HU was shown to raise fetal hemoglobin (HbF, α_2_γ_2_) levels in sickle cell disease (SCD) in 1985 [4], and it is now apparent that the requisite increase in γ-globin expression occurs via multiple mechanisms including, at a minimum, erythropoietic cytotoxicity leading to “stress erythropoiesis” with increased HbF levels, nitric oxide production, and the soluble guanylyl cyclase and cGMP-dependent protein kinase pathway [2]. Continued clinical research culminated in a Phase 3 double-blind randomized controlled trial published in 1995 [5] clearly establishing the utility of the drug in reducing the severity of sickle cell anemia (SCA). It also inhibits replication of human immunodeficiency virus-1 (HIV-1) [6] and has been used in cyanotic congenital heart disease [7]. The efficacy of HU for these varied clinical conditions appears to be due, at a minimum, to its potent inhibition of ribonucleotide reductase, a ubiquitous intracellular enzyme that converts ribonucleotides to deoxyribonucleotides, which are required for DNA synthesis and repair [8].

Although concerns have been expressed for potential tumorigenicity (especially leukemogenicity) of HU in the treatment of MPNs, analysis of these concerns has been confounded by the inherent leukemogenicity of these diseases. More recent retrospective and prospective analyses of long-term hydroxyurea use in the MPNs have been more reassuring regarding the drug’s previously alleged potential for fomenting malignant transformation [9-11]. In the non-malignant setting of SCD, too, data on the safety of long-term HU use have been similarly reassuring [12-16].

HU has been used in the treatment of mastocytosis, too, and has been associated with reductions in MC load and symptoms [17] as well as reductions in symptoms without clear reduction in MC load (e.g., malaise and pruritus [18]; progressive decrease in clinical symptoms and the need for intensive antimediator therapy [19]; weight loss, severe night sweats, abdominal pain, and pruritus [20]; facial flushing and bone pain [21]; and pruritus, flushing, ascites, and hepatosplenomegaly [22]). These reports suggest HU might be able to modulate MC mediator expression in some patients independent of its anti-proliferative effects.

Reported here are five cases of successful use of HU in MCAS to reduce symptoms. Each patient was diagnosed with MCAS after prior extensive evaluations failed to find any evident disease better explaining the full range of findings in the case and upon finding symptoms, physical exam findings, and laboratory evidence consistent with chronic aberrant MC mediator expression [1]. All five patients responded to assorted sets of MCAS-directed therapies. Of note, in all five patients modest doses of HU promptly, markedly reduced their diffuse aching, though cytopenias required stopping the drug in one patient, and reactions to one formulation of the drug in another three patients required trials of an alternative formulation.

### Case 1

A 55 year old white male airline pilot was referred in June 2009 for further evaluation of non-palpable splenomegaly discovered in the evaluation of chronic, waxing/waning nausea and left-sided abdominal pain, non-bloody emesis, early satiety, and mild weight loss that had emerged in the wake of an episode of alleged food poisoning in 2005. His flight privileges were revoked upon discovery of the splenomegaly, and he took a part-time retail sales position. Past history was notable for persistent problems with episodic diffusely migratory musculoskeletal pain complaints since unexplained left shoulder bursitis at age 9; repeated evaluations of these episodes were non-diagnostic. In addition to his gastrointestinal/abdominal symptoms, review of systems also revealed a sense of frequent variation in body temperature, sometimes even with mild rigors; near daily night sweats; episodic diffusely migratory edema; pruritic erythematous rash about the inferior neck; occasional acute spells of severe diffuse pruritus; waxing/waning dysgeusia and dysosmia; chronic tinnitus; chronic fatigue to the point of inability to get out of bed on some mornings; unpredictable/unprovoked acute-onset episodes every few days to every few weeks of lightheadedness and flushing; frequent palpitations; chronically irritated eyes; marked gastroesophageal reflux (intolerant of esomeprazole but improved with ranitidine); chronic back pain and diffusely migratory polyarthritis; alternating diarrhea and constipation; poor healing; and occasional diffusely migratory tingling/numbness paresthesias.

Examination was notable only for tenderness to palpation across the upper abdomen (worst in the epigastrium) and the above-noted neck rash but without pruritic behaviors. Small cherry angiomata were sparsely scattered about his skin. Moderate dermatographism was noted.

Serum tryptase was normal. Upon finding an elevated urinary prostaglandin D_2_ (PGD_2_) level during an “attack” plus increased (but non-aggregated, non-spindled) MCs by bright CD117 immunohistochemical staining in multiple gastrointestinal (GI) tract mucosal biopsies, MCAS was diagnosed. He gained incremental improvement with loratadine and famotidine and then aspirin, then quickly proved intolerant of serial trials of clonazepam, lorazepam, doxepin, quercetin, ketotifen, cromolyn, montelukast, and low-dose imatinib (200 mg daily), and then gained further improvement with low-dose dasatinib at 40 mg daily. Waxing/waning, diffusely migratory soft tissue and bone pain (typically 7/10 or worse) persisted without improvement, often was disabling, and proved refractory to a wide variety of analgesics prescribed by his local physicians. Hydrea-brand HU was begun in December 2011, initially at 500 mg daily, escalating weekly.

He returned in February 2012, having reached the prescribed target dose of 1500 mg daily. He reported the drug had initially worsened his nausea, abdominal discomfort, diarrhea, headache, and malaise, but after a week these symptoms completely resolved and his diffuse soft tissue pain completely resolved, too. He was able to stop aspirin and other occasional use of non-steroidal anti-inflammatory drugs (NSAIDs).

Two weeks later he reported new dyspnea. Thorough cardiopulmonary evaluation was unrevealing. Dasatinib was stopped.

In March 2012 soft tissue pain mildly relapsed, and HU had to be reduced to 500 mg daily due to excessive cytopenias. Fatigue, bone pain, headaches, palpitations, and diarrhea quickly relapsed, but there was insufficient improvement in cytopenias and the drug was fully stopped in April 2012. Dasatinib was restarted at 40 mg daily. Two weeks later he reported moderate improvement in many symptoms, but soft-tissue and bone pain continued. By August 2012 he was having so much trouble attending to his work due to his pain that he was considering applying for disability. Droxia-brand HU was tried at 200 mg daily, but it seemed to persistently exacerbate his left upper quadrant abdominal pain and was stopped after a month.

A trial of lorazepam compounded with baby rice cereal proved helpful for his soft tissue and bone pain. As of August 2013 he was well most of the time and controlling occasional flares with extra antihistamines and lorazepam. He was attending well to his part-time job and was reapplying for flight privileges.

### Case 2

A 29 year old disabled male manufacturing supervisor presented in August 2010 for further evaluation of a wide spectrum of chronic idiopathic problems dominated by diffusely migratory soft-tissue pain. Other than neonatal pulmonic valve repair and a number of accidental traumas in childhood, his early medical history was unrevealing, but at age 14 migraine headaches with attendant cognitive dysfunction (“brain fog”) suddenly developed and had persisted, waxing and waning, ever since. The headaches had often been attributed to chronic sinusitis, but the many sinusitis treatments he had tried had been uniformly unhelpful. By age 17 he was sleeping through many classes because classroom lights sometimes triggered headache flares. He suffered four more major accidental traumas at ages 17–18, once requiring resuscitation. He worked for a decade after finishing high school but had been unemployed since age 28 due to chronic right shoulder and back pain arising from a work-related accident. He also noted marked bilateral lower extremity pain had emerged in the months after the accident and was also attributed to this accident, although the only finding on a lumbosacral magnetic resonance imaging (MRI) scan was old right S1 pedicle trauma. His chief complaint was diffusely migratory pain, worst in the legs as a crampy, achy throbbing. Lorazepam and clonazepam tended to help his pain noticeably more than traditional analgesics, but his physicians were reluctant to regularly prescribe benzodiazepines.

On review of system he also noted severe gastroesophageal reflux for the prior decade, with frequent non-bloody post-prandial emesis. Esomeprazole helped only modestly. Spicy foods reliably triggered the reflux. Bidirectional endoscopy in 2004 to investigate an unrepeated episode of rectal bleeding was non-diagnostic. Episodic migratory swelling of his bilateral feet, ankles, and hands was also noted. Other problems included many years of intermittent subjective and objective idiopathic fevers, frequent night sweats, occasional chills, episodic diffusely migratory pruritus, eye and throat irritation, intranasal sores, deteriorating dentition, proximal dysphagia, waxing/waning dyspnea, palpitations, panic attacks, presyncope, syncope, diarrhea alternating with constipation, tinnitus, insomnia, poor healing, and diffusely migratory tingling/numbness paresthesias. Extensive evaluations by a variety of specialists had been unrevealing. He denied medication allergies. Past medical history included hypertension. He had not used illegal substances since adolescence.

Exam was notable for fatigue, cane-assisted ambulation, and obvious whole body discomfort with any motion. His skin was diffusely freckled. There were small lipomas scattered about his body which he noted had been progressing in number for several years. There was diffuse mild abdominal tenderness to deep palpation and 4/5 strength in all distal extremities; reflexes were intact. Moderate dermatographism was noted.

He appeared diffusely inflamed, but no specific inflammatory ailment had been identified in years of evaluation. Normal bone densitometry was noted. Porphyrin screening was negative. Serum tryptase was normal. In addition to mild relative eosinophilia, elevations in plasma PGD_2_ and histamine were found. MCAS was diagnosed. Loratadine and famotidine immediately provided major improvements in reflux, dyspnea, fevers, pruritus, and eye and nasal irritation. A one-month trial of celecoxib was moderately helpful for pain, but access to the drug could not be maintained. Serial trials of montelukast and azathioprine provided no additional benefit; trials of doxepin and low-dose imatinib quickly proved intolerable. Lorazepam proved helpful to varying degrees for sleep, malaise, anxiety and panic attacks, fevers, sweats, and palpitations but only mildly helpful for his diffuse pain.

In August 2012 HU was begun at 500 mg daily. For his first four-week supply, his pharmacist provided him two-week supplies of formulations from PAR and Barr. In the first two weeks using the Barr formulation, his leg pain was significantly improved, but three days after switching to the PAR formulation, all of his symptoms markedly worsened. Emergency room evaluation was unrevealing. He stopped HU and soon returned to his prior baseline. HU was then tried again with Droxia 200 mg capsules, which immediately reduced his diffuse pain from a persistent 10/10 at baseline to a sustained 6/10. No further improvement was seen at 200 mg twice daily dosing, but at 400 mg twice daily dosing, pain was substantially further reduced to 3/10. There was no hematologic toxicity. As of July 2013 he reported feeling comfortable and did not need further adjustments to his regimen.

### Case 3

In February 2011 a 55 year old disabled male businessman was referred for further evaluation of anemia and thrombocytopenia. He reported a lifelong history of multisystem polymorbidity of a generally inflammatory theme, including frequent upper and lower respiratory tract infections and “lots of diarrhea” ever since infancy. His parents were often upset with him about his illnesses and often suspected they were factitious. He also had been afflicted since his 20s by chronic diffusely migratory bone and joint and back pains. Chronic headaches and fatigue emerged in his 30s, and by his mid-40s severe gastroesophogeal reflux had emerged that proved refractory to aggressive medical and surgical therapies. On review of systems he endorsed fevers, chills, soaking sweats, diffusely migratory pruritus, spontaneous bruising, irritation of his eyes/nose/mouth/throat, hoarseness, cough occasionally productive of clear or colored sputum, dyspnea, chest pain requiring frequent emergency room evaluations which were always unrevealing, palpitations, proximal dysphagia, nausea, bloody and non-bloody vomiting, alternating diarrhea and constipation, urinary hesitancy and frequency, dysuria, diffusely migratory macular erythematous rash, diffusely migratory tingling/numbness paresthesias, diffusely migratory edema, poor healing, cognitive dysfunction, depression, presyncope, syncope, and panic attacks. In spite of all of these problems he had run a business for many years before progressive ailment disabled him at age 50. Past medical history included hypertension, hyperlipidemia, and pulmonary sarcoidosis in ‵87 treated with a right upper lobectomy; he had been dealing with a “low-grade” *Mycobacterium avium intracellulare* infection ever since and had been alternately told that possibly this or chronic aspiration might be causing many of his problems. The family history was rife was assorted cancers. His only known medication allergy was a penicillin-induced rash.

Exam was notable for a chronically ill, mildly diaphoretic general appearance, diffusely achy movement, and mild tenderness to palpation across the upper abdomen. Strong dermatographism was noted. He had had a mild normocytic anemia and mild thrombocytopenia for only the prior year; leukocyte count was normal, but eosinophils were 16%. Very extensive prior evaluations by multiple specialists had been unrevealing. Immunoglobulin G and M levels were normal, but IgA was found severely deficient. Sputum stains and cultures were negative, including testing for acid-fast bacilli. Serum tryptase was normal. Elevations in serum and urinary PGD_2_ were found. MCAS and IgA deficiency (more likely secondary to MCAS in view of his prior tolerance of red blood cell transfusions) were diagnosed.

Histamine H1 and H2 blockers were unhelpful. A trial of anti-inflammatories was desired, but aspirin and other traditional NSAIDs were felt to be contraindicated. Celecoxib lessened his fatigue, flushing, bruising, irritability, and cough. Low-dose cromolyn significantly reduced his abdominal discomfort and GI symptoms. Doxepin was unhelpful. Ketotifen further improved his energy. Low-dose lorazepam, too, further helped his malaise and GI symptoms, but diffusely migratory soft-tissue, bone, and joint pain remained a chief complaint.

In February 2013 Hydrea-brand HU was begun at 500 mg daily. In March 2013 he reported his bone pain was reduced; HU was increased to 1000 mg daily. In April 2013 he reported his bone pain had become tolerable. In May 2013 he stopped HU due to concerns it might interfere with healing from Mohs surgery, and his bone pain fully relapsed within a few days. He was unsure whether he was fully tolerating Hydrea. Droxia-brand HU was begun at 200 mg daily. His constant 8/10 bone pain immediately decreased to 2/10, and his cough and sputum production almost completely resolved, too. There was no hematologic toxicity. His muscle pain, though, was unimproved, and he was planning to try increasing his Droxia.

### Case 4

In January 2012 a 30 year old female laboratory technologist was referred for further evaluation of suspected mast cell disease. She had been healthy until an emergency Cesarean section was required at age 19 due to infection, after which she developed generalized weakness and bilateral lower extremity bone and joint pains which never resolved. At age 24, shortly after her father died, significant alopecia and chronic fatigue emerged, and the chronic aching extended to involve her hands, too. In 2008 her fatigue was assumed to be due to obstructive sleep apnea for which she underwent tonsillectomy and septoplasty, the only apparent result of which was worsening of fatigue and joint pains. Multiple rheumatologic and neurologic evaluations were negative except for tentative conclusions (based on modest elevations in ANA) of lupus for which trials of Plaquenil and methotrexate never yielded any discernible improvement. “Cigarette-burn-like” rashes sometimes like hives and often leaving scars, together with diffusely migratory pruritus, emerged at age 29. Joint pains came to involve her elbows, largely incapacitating her use of her arms. Frequent nausea and diarrhea alternating with constipation emerged, too, but extensive gastroenterologic evaluation was negative. On review of systems, she also endorsed waxing/waning issues subjective fevers, chills, soaking sweats, flushing, diffusely migratory marked aching, dysmenorrhea, headaches, irritated eyes, frequent coryza, irritated mouth, mild dyspnea, proximal dysphagia, palpitations, refractory gastroesophageal reflux, diffusely migratory edema, diffusely migratory tingling/numbness paresthesias, spontaneous bruising, episodic cognitive dysfunction, and daily presyncope. There were multiple cancers in the family history. Her only known allergy was a sulfa-induced rash.

Exam found a tired, overweight, achy woman with a sparse scattering of small macular dark or lightly erythematous lesions, slight tenderness at all nodal stations, and an abdomen notable for diffuse mild tenderness and diaphoresis, and trace distal edema. Moderately bright dermatographism was noted. Serial complete blood counts were notable only for a stable borderline microcytosis, chronic mild leukocytosis (with frequent minimal monocytosis and/or eosinophilia). Alkaline phosphatase was chronically minimally elevated. Anti-nuclear antibodies were mildly elevated, C-reactive proteins were persistently significantly elevated, and erythrocyte sedimentation rates were normal. Extensive thyroid testing was normal.

Serum tryptase was normal, but plasma histamine and urinary PGD_2_ were found elevated (this last in spite of ongoing, if ineffective, use of ibuprofen). MCAS was diagnosed. Loratadine and famotidine immediately resolved her rash. Aspirin, lorazepam, doxepin, quercetin, and cromolyn were unhelpful. Montelukast 10 mg twice daily (but not once daily) improved her fatigue and emotional lability.

In April 2013, in view of her diffusely migratory pain being her chief complaint, Hydrea-brand HU was initiated at 500 mg daily. This immediately began helping her pain and nausea. She previously had needed to often recline and take weight off her legs due to throbbing pain throughout her legs (typically 8/10 or worse), but HU allowed her to stay upright throughout the day. These improvements were amplified upon increasing the dose to 1000 mg daily and were sustained as of July 2013 (pain score 3/10). There was no hematologic toxicity.

### Case 5

In October 2010 a 62 year old retired law enforcement officer was referred for further evaluation of heterozygous alpha-1-antitrypsin deficiency discovered while being evaluated for mysterious chronic debilitating illness. Crohn’s disease had been diagnosed by colonoscopic biopsy in 2003. Mesalamine was unhelpful for his diarrhea. Repeat endoscopy in 2009 led to a change in diagnosis to lymphocytic colitis. Budesonide resolved his diarrhea. Several months later (one month after a trip), a left lower extremity deep venous thrombosis and bilateral pulmonary emboli developed. Chronic anticoagulation was begun. He began rapidly losing weight. Budesonide was stopped. Diarrhea did not immediately relapse. Past history included chronic back and bilateral leg pain since a work-related fall in 1967. On review of systems, he endorsed chronic fatigue, anhedonia, depression, lethargy, feeling cold all the time since starting warfarin, heat intolerance, diffuse aching since 2009, tinnitus, constant coryza, throat irritation, dry cough, constant hunger, constipation (possibly from chronic oxycodone use), urinary frequency, frequent confusion, easy irritability, and frequent presyncope. He was an active smoker with a 100-pack-year cigarette history.

Exam found a thinning, worried man with livedo reticularis across the low back and mild dermatographism. Extensive prior laboratory testing was notable only for stable mild elevations in hepatic transaminases. The serum ferritin was twice the upper limit of normal. Liver biopsy showed him to be heterozygous for alpha-1-antitrypsin deficiency but did not show alpha-1-antitrypsin globules.

Serum tryptase was normal. Plasma PGD_2_ was double the upper limit of normal. Factor VIII was markedly elevated at 400% (normal 50-150%). CD117 immunohistochemical staining of endoscopic biopsies from 2009 showed significantly increased (though non-aggregated, non-spindled) mast cells. MCAS was felt likely, possibly with a comorbid Factor VIII-overexpressing hypercoagulable state, though given that Factor VIII is a known MC mediator, his hypercoagulable state could have been secondary to his MCAS.

He started loratadine and famotidine and soon noted mild to moderate improvement in almost all symptoms, but diffusely migratory aching was unimproved. Montelukast was unhelpful. Aspirin further helped many of his symptoms but, again, not the aching. Low-dose lorazepam mildly reduced his pain. Doxepin was unhelpful. Cromolyn essentially resolved his GI symptoms. Low-dose imatinib (200 mg daily) reduced his fatigue and cognitive dysfunction and finally provided him “more good days than bad,” but diffuse aching was unimproved, typically 8/10 or worse.

In February 2013 Hydrea-brand HU was initiated at 500 mg daily but immediately proved intolerable (nausea, vomiting, chills). In April 2013 Droxia-brand HU was initiated at 200 mg daily. In June 2013 he reported significant reduction in not only fatigue but also diffuse aching (3/10) and presyncope. There was no hematologic toxicity. As of July 2013 he was planning to try escalating the Droxia dose.

## Discussion

Of hematopoietic origin, mast cells (MCs) are found in all human tissues, especially at the environmental interfaces and perivascular/perineural sites [23]. They serve largely as sentinels of environmental change and bodily insults and respond by releasing large and variable assortments of molecular mediators which directly and indirectly influence behavior in other (local and distant) cells and tissues to respond to changes/insults so as to maintain, or restore, homeostasis. The transmembrane tyrosine kinase receptor KIT is the dominant MC regulatory element, shown to be critical for key MC functions including survival, differentiation, chemotaxis, and activation [24].

Traditionally, MC disease has been thought to be principally a matter of neoplastic burdens of MCs, with symptoms resulting principally from an accompanying inappropriate release of mediators from these excessive numbers of MCs. Nearly a quarter century ago, though, the notion was first advanced that there might be forms of MC disease manifesting inappropriate mediator release with little to no accompanying proliferation of MCs [25]. This theory appeared validated when the first recognized cases of what is now called MC activation syndrome (MCAS) were published in 2007 [26-28]. Different patterns of aberrant expression of the large MC mediator repertoire in different MCAS patients make for markedly heterogeneous – and thus diagnostically challenging – presentations [1]. The cause of such heterogeneity is not yet clear. Provocatively, though, Molderings *et al.* have repeatedly found a wide array of (presumably mostly constitutively activating) mutations scattered across all domains of KIT in small cohorts of MCAS patients, with most of their studied patients bearing multiple mutations in no yet-apparent recurring patterns [28,29]. (Interestingly, the KIT^D816V^ mutation so common in mastocytosis seems rare in MCAS.) Though these findings have not yet been independently confirmed, it is noteworthy that similar mutational complexity (in KIT and other cellular controllers) has been found, too, across the spectrum of chronic myeloproliferative neoplasms (MPNs) within which the MC disorders reside [30], and in advanced mastocytosis itself [31].

Given these new biologic and clinical insights, proposals have emerged to consider all MC diseases under the umbrella term of MC activation disease (MCAD) [32]. It also has been proposed that the assorted systemic MCAD variants and clinical phenotypes represent not distinct disease entities but instead varying presentations of a common generic root process of mast cell dysfunction [33]. Mastocytosis may be merely the tip of the proverbial MCAD iceberg, fairly readily recognizable (in spite of its rarity) because of its distinctive clinicopathologic presentation, while the bulk of the iceberg – hidden below the waterline of easy clinical recognizability – may be a far larger, and far more heterogeneous, collection of variants of MCAS, some of which are already discretely recognized (e.g., idiopathic anaphylaxis [27], cryopyrin-associated periodic syndrome [34]), but most not.

With no predictors of therapeutic response yet identified, MCAD’s heterogeneity poses not only diagnostic but also therapeutic challenges (Table 1). The cases in the present series extend earlier observations that HU appears able to modulate MC mediator expression in MCAD, including MCAS, and may be particularly useful to treat pain in patients refractory to, or intolerant of, other typical and atypical analgesics. Although all of the patients reported here were already on some anti-mediator therapy at the time HU was begun, the fact that initiation of HU was the only medication change preceding the observed further symptomatic improvements strongly suggests HU was solely responsible for these improvements.

**Table 1:** Treatments used in mast cell activation disease

**Mechanism of action**	**Therapies / therapeutic classes**
Anti-mediator (inhibition of MC mediator production or action)	Histamine H_1_ receptor antagonists
	Histamine H_2_ receptor antagonists
	Non-steroidal anti-inflammatory drugs
	Benzodiazepines
	Corticosteroids
	Leukotriene receptor antagonists
	Proton pump inhibitors
	Tricyclic antidepressants (antihistaminic)
	Phenothiazines (antihistaminic)
	Hydroxyurea
	Bisphosphonates, vitamin D, calcium
	5-hydroxytryptamine_3_ receptor antagonists
	Ivabradine
	Icatibant
	Alpha lipoic acid
	N-acetylcysteine
	Amphetamines
	Hypolipidemics
Mast cell stabilizers	Cromolyn
	Quercetin
	Ketotifen
	Pentosan
	Tyrosine kinase inhibitors
	Alpha interferon
	Vitamin C
Cytostatic/cytotoxic agents	Cladribine
	Pentostatin
	Fludarabine
	Paclitaxel
	Alkylators (e.g., cyclophosphamide)
	Hydroxyurea
Immunomodulation	Allergy desensitization therapy
	Allogeneic stem cell transplantation

Three of the patients in this series quickly showed intolerance of a commonly used formulation of HU but then tolerated, and responded well to, an alternative formulation, possibly due to the propensity of MCAD patients to manifest odd and prolific medication sensitivities which may result from MC reactions to either “active” or “inactive” ingredients. It may be worthwhile when designing therapeutic trials for the MCAD population to allow for switching to alternative medication formulations when intolerance of a given formulation quickly becomes apparent. Such a strategy may reduce the chance of underestimating tolerability and efficacy.

The clinical observations from the present case series may gain further significance in light of the recent discovery by Vincent *et al.* that in a murine model of sickle cell anemia (SCA), MC activation was found to underlie sickle pathophysiology leading to inflammation, vascular dysfunction, pain, and requirement for high doses of morphine [35]. HU is used as a disease-modifying agent in sickle cell disease (SCD) and is the only approved such agent, though red blood cell transfusions, too, can greatly decrease disease severity and hematopoietic stem cell transplantation can cure SCD [36]. Despite use of HU in this fashion for more than 25 years, the full set of mechanisms by which it reduces SCD severity remains unclear. The efficacy of HU in the treatment of SCD is generally attributed to its ability to increase HbF, but in a recent systematic review of the efficacy of HU in SCD, the sole identified randomized controlled trial reported the mean increase in HbF from two years of HU use was only 3.2% [37], while other salutary benefits of HU in SCD not clearly related to increases in HbF have been observed, too, including lowering of circulating leukocyte counts and local release of nitric oxide [2]. It is unclear whether HU’s established activity as a ribonucleotide reductase inhibitor is the sole molecular mechanism of action through which all of these benefits arise.

Thus, given the recent data from Vincent *et al.*, the overlap in the patterns of pain in both SCD and MCAS, estimates of significant (14-17%) prevalence of MCAS in the general population [33,38], and the observations from the present case series of the utility of HU in the management of otherwise refractory pain in some MCAS patients, at least two additional questions are raised: (1) might unrecognized MCAS account for some of the comorbidities which have long been seen in some SCD patients, and attributed to their SCD, but for which it has been difficult identifying specific biologic pathways supporting such attribution, and (2) might treatment of unrecognized MCAS be another mechanism by which HU helps in SCD?

## Conclusions

MCAS should be considered in the differential diagnosis of diffusely migratory soft tissue, bone, and/or joint pain. HU appears useful in treating an assortment of mediator-driven symptoms in some patients with MCAS. In particular, HU may be useful in addressing otherwise refractory soft tissue and/or bone pain in MCAS, but further study is needed to define HU’s mechanisms of action in MCAD/MCAS, optimal dosing strategies, and which patient subpopulations are most likely to benefit. Further study may also be warranted to characterize the prevalence and behavior of MCAS in SCD patients, particularly those with comorbidities not easily attributable to erythrocyte sickling.

## Consent

Written informed consent was obtained from each patient for inclusion in publication of this case series. Copies of the written consents are available for review by the Editor-in-Chief of this journal.

## Abbreviations

GERD: Gastroesophageal reflux disease; IgA: Immunoglobulin A; HU: Hydroxyurea; MC: Mast cell; MCAD: Mast cell activation disease; MCAS: Mast cell activation syndrome; MPN: Myeloproliferative neoplasm; MSK: Musculoskeletal; pPGD2: Plasma prostaglandin D_2_; uPGD2: Urinary prostaglandin D_2_; SCD: Sickle cell disease.

## Competing interests

Dr. Afrin reports he has no conflicts of interest, received no support for this work, and had full access to all of the data in the study and takes responsibility for the integrity of the data and the accuracy of the data analysis. This work has not been presented previously in any other form or venue.

## Author’s information

LBA is an Associate Professor of Medicine in the Division of Hematology/Oncology at the Medical University of South Carolina in Charleston, SC, USA.

## Support

No funding or other support was received for this work from any source.
